# Fusion Domain-Adaptation CNN Driven by Images and Vibration Signals for Fault Diagnosis of Gearbox Cross-Working Conditions

**DOI:** 10.3390/e24010119

**Published:** 2022-01-13

**Authors:** Gang Mao, Zhongzheng Zhang, Bin Qiao, Yongbo Li

**Affiliations:** MIIT Key Laboratory of Dynamics and Control of Complex System, School of Aeronautics, Northwestern Polytechnical University, Xi’an 710072, China; mg0207@yeah.net (G.M.); zhangzhongzheng@mail.nwpu.edu.cn (Z.Z.); qiaobin@mail.nwpu.edu.cn (B.Q.)

**Keywords:** deep learning, fault diagnosis, multi-source heterogeneous fusion, gearbox, transfer learning

## Abstract

The vibration signal of gearboxes contains abundant fault information, which can be used for condition monitoring. However, vibration signal is ineffective for some non-structural failures. In order to resolve this dilemma, infrared thermal images are introduced to combine with vibration signals via fusion domain-adaptation convolutional neural network (FDACNN), which can diagnose both structural and non-structural failures under various working conditions. First, the measured raw signals are converted into frequency and squared envelope spectrum to characterize the health states of the gearbox. Second, the sequences of the frequency and squared envelope spectrum are arranged into two-dimensional format, which are combined with infrared thermal images to form fusion data. Finally, the adversarial network is introduced to realize the state recognition of structural and non-structural faults in the unlabeled target domain. An experiment of gearbox test rigs was used for effectiveness validation by measuring both vibration and infrared thermal images. The results suggest that the proposed FDACNN method performs best in cross-domain fault diagnosis of gearboxes via multi-source heterogeneous data compared with the other four methods.

## 1. Introduction

The gearboxes play an irreplaceable role in the mechanical power system, which usually works in harsh and complex environments [1,2]. The failure of gearboxes may cause unexpected accidents and economic losses. Therefore, accurately identifying and diagnosing faults is the key to ensuring that gearboxes normally operate [3]. 

The intelligent diagnosis method has received wide attention from researchers because of its ability to detect faults automatically and is not limited by manual experience. The methods based on deep learning are particularly prominent because they can adaptively learn the fault information hidden in the collected signals, such as long short-term memory network (LSTM) [4], recurrent neural network (RNN) [5] and convolutional neural network (CNN) [6]. In addition, some extended models based on standard deep learning models are proposed for rotating machinery fault diagnosis, such as deep convolutional auto-encoder (DCAE) [7], CNN with capsule network [8] and multiscale CNN [9], etc. Yao et al. [10] proposed a stacked inverted residual CNN (SIRCNN), which had stable and reliable fault diagnosis accuracy. Shao et al. [11] established an ensemble deep auto-encoder (EDAE), which consists of several DAEs with different activation functions. The results indicated that it has good accuracy in rolling bearing fault diagnosis. However, the methods mentioned above assume that adequate high-quality data collected from the concerned machine are available for estimating underlying data distributions. In addition, these methods need training and testing data drawn from the same probability distribution [12]. In actual applications, it is impractical to obtain a large amount of labeled data. In addition, the performance of the aforementioned methods may decrease in recognizing unlabeled data collected from another machine or different working conditions due to data discrepancy [13].

Transfer learning can transfer learned knowledge to related machinery or fields, and it is widely applied to address the above-mentioned cross-domain fault diagnosis problem. Currently, parameter transfer and feature domain-adaptation are two popular transfer learning implementation methods. Parameter transfer is suitable for scenarios where only a few labeled data from the target domain are available, but it is not enough to train the model. Qian et al. [14] proposed a method for rolling bearing fault diagnosis under variable working conditions by transferring the parameters of the stack auto-encoder (SAE). Chen et al. [15] proposed the transfer neural network to diagnose the faults of the rotary machinery, which pre-trains a 1D-CNN with the source data and then uses the limited target data to fine-tune the model to obtain a transfer convolutional neural network. However, in most practical applications, there is no available labeled target data to participate in the model training process. Domain adaptation techniques based on feature transfer have been much preferred in this case. One implementation of domain adaption is to add a domain adaptation term to the loss function, such as Maximum Mean Discrepancy (MMD) [16,17,18] and Wasserstein distance [19]. Another implementation of domain adaption is through domain adversarial training, in which a feature extractor aims to extract common features from both source and target domain by adversarial training [20,21,22]. In addition to this, in order to further improve transfer and generalization capabilities of the models, multiple source domains of data are used to extract transferable features, which are used to diagnose the faults of rotating machinery [23,24,25]. 

However, most existing studies on transfer diagnosis mainly focus on single-channel signals with vibration signals as the mainstay. This is because the vibration signal can be collected by the acceleration sensor attached to the surface of the component, which is sensitive to the impact caused by structural damage, such as gear fracture and bearing outer race crack. For some non-structural faults, such as gear box oil shortages, vibration signals are not sensitive to them. These failures can also cause serious consequences and should not be ignored. Infrared thermal image can perfectly reflect non-structural fault information and is widely applied in fault diagnosis [26,27]. However, the single infrared thermal image is very sensitive and is easily affected by external factors such as oil temperature [28]. Therefore, the fault diagnosis method based on multi-source heterogeneous data fusion is an issue worthy of study. Bai et al. [29] proposed a method for coupling fault diagnosis of rotary machinery by using infrared images and vibration signals, in which the enhanced infrared thermal image and two-dimensional vibration signals are spliced and inputted into CNN to obtain final diagnosis result. Shao et al. [30] pre-trained multiple novel SAEs using multisensory signals from the source domain and finetuned each novel SAE using a target domain sample. The diagnosis result is obtained by a modified voting strategy. In the above research studies, multi-source heterogeneous signals are widely applied in fault diagnosis since they can supply abundant fault information. However, it is rare to use infrared thermal images and vibration signals to diagnose structured and unstructured failure states in unlabeled target domains. 

This paper proposed a fusion domain-adaptation CNN (FDACNN) driven by images and vibration signals. An FDACNN consists of two main stages: data-level fusion and domain-adaptation network training. In the stages of data-level fusion, raw signals are transformed into frequency and squared envelope spectrum, and they are arranged into two-dimensional format. Two-dimensional format data are combined with the infrared thermal image to form fusion data samples for model training. In the stages of domain-adaptation network training, a features extractor, a domain discriminator and a state classifier are constructed. After a number of adversarial training, the domain invariant features can be extracted from fusion samples and used for the classification of health states. In actual industrial gearbox, both the accelerators and infrared camera can be installed to collect the infrared images and vibration signals. The vibration signal can be used to effectively diagnose structural failures such as tooth breakage, tooth missing, and gear wear. Moreover, the infrared thermal image is sensitive to non-structural failures, such as oil shortage and oil temperature exorbitant. In this study, more comprehensive features can be extracted from infrared thermal image and vibration signals than a single sensor. Moreover, the proposed method has lower calculation complexity, which can rely in the online fault diagnosis of gearbox. The main contributions and insights of this study are listed below:

(1) A data-level multi-source heterogeneous fusion scheme is proposed. The frequency and squared envelope spectrum can more clearly reflect fault information contained in the vibration signal. The fusion of the preprocessed vibration signal and the infrared thermal image makes the fault information in the training sample more abundant and obvious.
(2) A fusion domain-adaptation CNN fault diagnosis method for gearboxes is explored. It can extract domain invariant features from the fusion information of vibration signals and infrared thermal images and implement gearbox fault diagnosis in an unlabeled target domain.

The rest of this article is arranged as follows: Section 2 presents preliminary and basic knowledge. The details of the proposed FDACNN are provided in Section 3. Section 4 validates the proposed method and analyzes the results. Finally, the conclusion in Section 5 brings the study to a close.

## 2. Preliminaries

### 2.1. Squared Envelope Spectrum

Demodulation analysis methods can extract and identify fault characteristic frequencies from the resonance band. Envelope analysis is widely applied to acquire the harmonics of characteristic frequencies, such as the squared envelope spectrum (SES). Since the faulty rotating machinery vibration signals usually contain second-order cyclostationary (CS2) components, they are often used to extract fault features. CS2 is commonly calculated using SES, and the formulas can be expressed as follows:

(1) $$SES(\alpha )={\left|\frac{1}{L}\sum_{n=0}^{L-1}{\left|\tilde{x}(n)\right|}^{2}e^{-j2\pi n\alpha /F_{s}}\right|}^{2}={\left|DFT\left({\left|\tilde{x}(n)\right|}^{2}\right)\right|}^{2}$$

where α represents cyclic frequency, and *F_s_* denotes the sample frequency. $\tilde{x}\;(n)$ is converted by the Hilbert transform from time-domain vibration signals. DFT(•) represents the discrete flourier transform, and it is formulated as follows:

(2) $$DFT[x(n)]=\sum_{n=0}^{L-1}x\;(n)\;e^{-j\cdot k\;n\frac{2\pi }{L}}$$

where x (n) represents the signal sequence [0 *L*−1]. Thus, CS2 components is acquired with cyclic frequency α.

### 2.2. Convolutional Neural Network

Convolutional neural network is a typical deep feed-forward artificial neural network that can be used to process time sequences and images by convolution operation. This operation can reduce the number of weights and biases to decrease the complexity of the model. A standard CNN consists of convolution layer, pooling layer, fully connected layer and classification layer. In a convolutional layer, multiple convolutional kernels are used to convolution the input, and the weights and bias are shared between hidden neurons. The process in the convolutional layer can be expressed as follows:

(3) $$z_{n}^{l}=f^{l}(\sum_{k}x_{k}^{l-1}*w_{n}^{l}+b_{n}^{l})$$

where $x_{k}^{l-1}$ is the *k*-th input sample in the *l*-1 layer. * represents convolution operation. $w_{n}^{l}$ and $b_{n}^{l}$ denote the weight and corresponding bias, respectively. Additionally, $f^{l}(\cdot )$ represents the activation function.

A pooling layer usually follows the convolutional layer, and the subsampling operations is employed to reduce the spatial dimension for reducing overfitting risk. Mathematically, a maximum pooling operation is defined as follows:

(4) $$po_{j}=\underset{i\in m_{j}}{\max\{c_{j}(i)\}}$$

where $c_{j}$ represent the *j*-th location, and $po_{j}$ is the output of the pooling. Moreover, average pooling and stochastic pooling are also usually used in pooling layer.

After several convolutional and pooling layers, the fully connected layer immediately converts the output matrix into a row or column. The last layer is usually served by a softmax output layer in which a softmax function is utilized to predict the probability of each target.

### 2.3. Deep Adversarial Convolution Neural Network

Generally, a deep adversarial convolution neural network usually consists of a feature extractor *G_f_*, a domain discriminator *G_d_* and a classifier *G_c_* [13]. The feature extractor, which is a competitor in the DACNN, is typically served by several convolution blocks or fully connected layers. It can be expressed as $G_{f}=G_{f}(x,\;\theta_{f}):\;x\to R^{D}$ with parameter $\;\theta_{f}$, which indicates that the input sample *x* is transformed into *D*-dimensional features. In addition, the domain discriminator (binary classifier) is treated as the opponent, which is expressed as $G_{d}=G_{d}(G_{f}(x),\;\theta_{d})$ with parameters $\;\theta_{d}$. Inputting the source and target samples into the feature extractor and the output is further distinguished by the domain discriminator *G_d_*. The binary cross entropy (BCE) loss is taken as objective function, which can be described as follows:

(5) $$L(G_{d}(G_{f}(x_{i})),d_{i})\;=\;d_{i}\;\log\frac{1}{G_{d}(G_{f}(x_{i}))}+(1-d_{i})\times \log\frac{1}{1-G_{d}(G_{f}(x_{i}))}$$

where *d_i_* denotes the binary variable for *x_i_*. By conducting adversarial training between the two parts, feature extractor *G_f_* tends to extract common features from two types of data and makes the domain discriminator difficult to distinguish in terms of zero or one. Thus, the model can perform well on both the source and target datasets. Assuming *n* samples in the source domain dataset and *N*-*n* samples in the target domain dataset, the objective function is expressed as follows:

(6) $$E(\theta_{f},\;\theta_{d})=-\;(\frac{1}{n}\sum_{i=1}^{n}L_{d}^{i}(\theta_{f},\;\theta_{d})+\frac{1}{N-n}\sum_{i=n+1}^{N}L_{d}^{i}(\theta_{f},\;\theta_{d}))$$

where $L_{d}^{i}(\theta_{f},\;\theta_{d})=L_{d}(G_{d}(G_{f}(x_{i},\;\theta_{f}),\;\theta_{d}),\;d_{i})$, and this equation includes a maximization problem with respect to $\theta_{d}$ and a minimization problem with respect to $\theta_{f}$.

Additionally, all the labeled samples should be supervised and trained to ensure the accuracy of the diagnosis in the adversarial procedure. Therefore, a classifier is established, and it is expressed as $G_{y}=G_{y}(G_{f}(x),\;\theta_{y}):R^{D}\to R^{L}$ with parameters $\theta_{y}$, in which *L* is the number of classes. Cross-entropy loss is applied in the Softmax function, and it can be described as follows.

(7) $$L_{y}(G_{y}(G_{f}(x_{i})),y_{i})=\log\frac{1}{G_{y}{\left(G_{f}(x_{i})\right)}_{y_{i}}}$$

By adding the Equation (7) to objective function (6), the optimization objective can be expressed as follows:

(8) $$E(\theta_{f},\;\theta_{y},\;\theta_{d})=\frac{1}{n}\sum_{i=1}^{n}L{\;}_{y}^{i}(\theta_{f},\;\theta_{y})\;-\lambda (\frac{1}{n}\sum_{i=1}^{n}L{\;}_{d}^{i}(\theta_{f},\;\theta_{d})+\frac{1}{N-n}\sum_{i=n+1}^{N}L_{d}^{i}(\theta_{f},\;\theta_{d}))$$

where $L_{y}^{i}(\theta_{f},\;\theta_{y})=L_{y}(G_{y}(G_{f}(x_{i},\;\theta_{f}),\;\theta_{y}),\;y_{i})$. The entire training process of DANN is to optimize the parameters $\theta_{f}$, $\theta_{y}$ and $\theta_{d}$, and it is expressed as follows.

(9) $$\left(\hat{\theta_{f}},\;\hat{\theta_{y}})=\underset{\theta_{f},\;\theta_{y}}{\mathrm{argmax}}E(\theta_{f},\;\theta_{y},\;\hat{\theta_{d}}\right)$$

(10) $$\hat{\theta_{d}}=\underset{\theta_{d}}{\mathrm{argmax}}E(\hat{\theta_{f}},\;\hat{\theta_{y}},\;\theta_{d})$$

In the training stage, parameter updates are implemented in the opposite direction to the gradient in the adversarial process. The update in the domain discriminator *G_d_* is to reduce the loss with the purpose of improving the discriminative ability. However, the update in the feature extractor *G_f_* is to maximize the loss to fool the discriminator. In order to frame a flexible implement of the stochastic gradient descent (SGD) algorithms in the training of DACNN, we use a circuitous method by rewriting the loss as $L^{\prime }{}_{d}^{i}(\theta_{f},\;\theta_{d})=L_{d}(G_{d}(G_{f}(x_{i},\;\theta_{f}),\;\theta_{d}),\;1-d_{i})$ in updating the feature extractor parameters. Maximizing $L_{d}^{i}(\theta_{f},\;\theta_{d})$ can be accomplished by minimizing $L^{\prime }{}_{d}^{i}(\theta_{f},\;\theta_{d})$. During backpropagation, the features extractor takes the gradient of the recalculated $L^{\prime }{}_{d}^{i}(\theta_{f},\;\theta_{d})$ from the domain discriminator and updates its parameters with SGD. Overall, the update rales of parameters $\theta_{f}$, $\theta_{y}$ and $\theta_{d}$ can be formulated as follows:

(11) $$\theta_{f}\leftarrow \theta_{f}-\mu \;(\frac{\partial L_{y}^{i}}{\partial \theta_{f}}+\lambda \frac{\partial L^{\prime }{}_{d}^{i}}{\partial \theta_{f}})$$

(12) $$\theta_{d}\leftarrow \theta_{d}-\mu \;\frac{\partial L_{d}^{i}}{\partial \theta_{d}}$$

(13) $$\theta_{y}\leftarrow \theta_{y}-\mu \;\frac{\partial L_{y}^{i}}{\partial \theta_{y}}$$

where μ represents the learning rate.

By the above optimization process, DACNN tends to train a feature extractor *G_f_* that can extract suitable representations from input samples (either source domain datasets or target domain datasets), which can be classified accurately by the classifier *G_y_* but weakens the ability of the domain discriminator *G_d_* to differentiate a sample from the source or target domain datasets. In the phases of testing, domain insensitive features will be extracted by feature descriptor *G_f_* and fed into the classifier *G_y_* to identify the states immediately.

## 3. The Proposed Method

### 3.1. Data-Level Fusion

Data-level fusion is a relatively direct fusion method that can retain the effective fault information hidden in the measured signals and reduce the complexity of the model. Therefore, a data-level fusion strategy is designed in this study to fuse one-dimensional vibration signals and two-dimensional infrared thermal images. 

**Figure 1:**
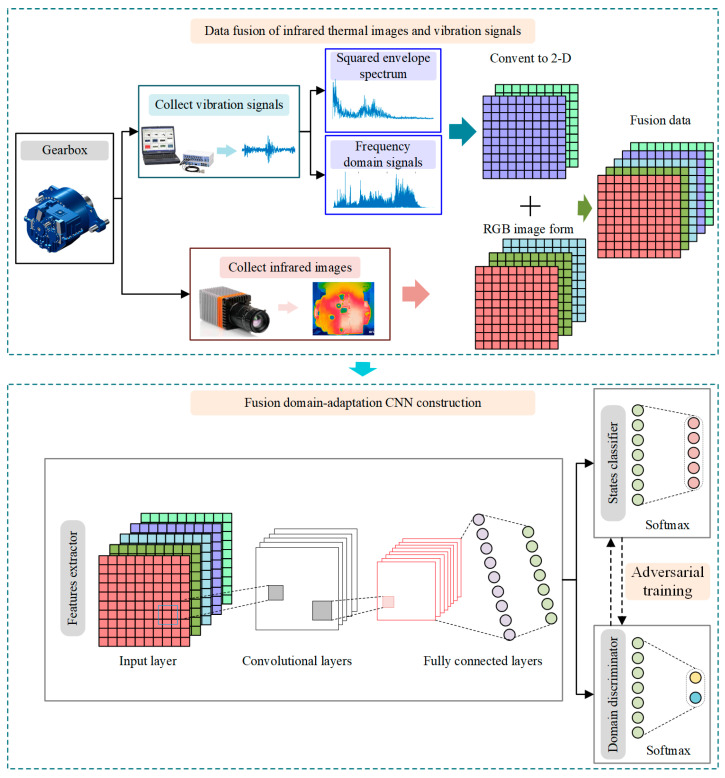
The procedures of the proposed method.

In general, the measured time-domain vibration signals are feeble and inadequate, particularly in the early stages of a failure. Although many deep learning methods only use raw time-domain signals for fault diagnosis, they rely heavily on deep learning structure. It is considered that frequency features play a significant role in rotating machinery failure diagnostics. The frequency domain signal and the squared envelope spectrum, in particular, are widely used in classical signal processing methods [31,32]. Therefore, measured raw signals are transformed to acquire frequency domain signals by fast Fourier transform (FFT) and CS2 by squared envelope spectrum in this study. Then, they are reshaped into two-dimensional matrix as part of the input of the convolution layer as shown in Figure 1.

After that, the RGB 3-channels of each infrared thermal image are combined with the 2-dimensional frequency domain signal and the squared envelope spectrum to synthesize a 5-channel data. The 5-channel fusion data will be used for subsequent domain adaptation CNN training. 

### 3.2. Fusion Domain-Adaptation CNN Construction

Domain adaptation based on adversarial networks is an effective approach for cross-domain fault diagnosis. In this section, fusion 5-channel data are used to train a domain-adaptation CNN for cross-domain fault diagnosis of gearboxes.

Firstly, a feature extractor is constructed, which contains multiple convolutional blocks and several fully connected layers. The feature extractor is used to extract domain-insensitive features from 5-channel fusion data. The extracted features are fed into the state classifier to recognize health states. Meanwhile, the extracted features are inputted into the domain discriminator to distinguish whether they are from the source or target domain.

In the training process, the feature extractor minimizes the state classification loss and maximizes the domain discrimination loss so that it can extract features that are not only not sensitive to the domain and make the state classifier easy to classify. As the opponent, the domain discriminator aims to minimize domain discrimination loss so that it can distinguish the feature from the source or target domain. Finally, through a large amount of adversarial training, the diagnosis model composed of feature extractor and state classifier can recognize fault states accurately with multi-source heterogeneous data under cross-working conditions.

### 3.3. Procedures of Proposed Fusion Domain-Adaptation CNN

This section presents the summaries of the proposed fusion domain-adaptation CNN as shown in Figure 1. The main procedures are described as follows.

Collect the infrared thermal images and raw vibration signals from the concerned gearboxes under different working conditions and divide them into labeled source domain samples and unlabeled target domain samples.

Convert the raw time-domain signals into frequency domain signals and the squared envelope spectrum and arrange them into matrixes.

Fuse the RGB 3-channels of infrared thermal image and two matrixes (frequency domain and squared envelope spectrum) to obtain 5-channel fusion samples.

Train the FDACNN model using 5-channel fusion samples by adversarial training.

Test the performance of the proposed FDACNN model by using the remaining samples from the target domain.

## 4. Experimental and Result Discussion

### 4.1. Dataset Descriptions

**Figure 2:**
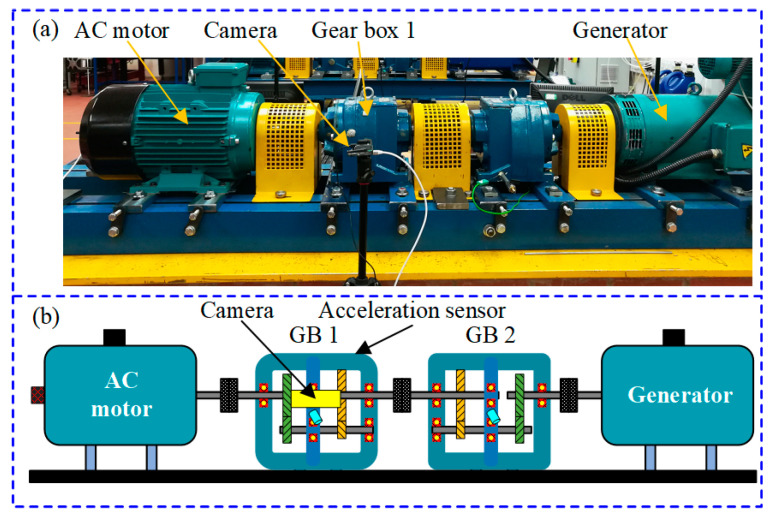
The gear box fault simulator system: (**a**) the experimental test rig; (**b**) the layout of the test rig.

Test data are from a compound gear failure experiment performed on a helical gearbox called Spectra Quest Mechanical Failure Simulator (MFS) from Northwestern Polytechnical University lab [33,34]. The experiment system and the layout of the experiment rig are shown in Figure 2a,b, respectively. The experiment system mainly consists of an AC motor, two gearboxes and a generator. The infrared camera is fixed on the front of gearbox 1 to collect the infrared thermal image. The detailed parameters of the infrared camera are listed in Table 1. Vibration signals are collected by an acceleration sensor mounted on the surface of the gearbox 1. The sample frequency is 12.8 kHz, and the motor speed is 3000 rpm. In this experiment, there are two kinds of lubricating oil, i.e., EP 320 and EP 100. EP 320 lubricant viscosity is 320cSt @ 40 °C, EP 100 lubricant viscosity is 100cSt @ 40 °C. The EP 320 lubricant is applied in this article. 

In this study, five different health states are introduced, including a normal state, two structural fault states (TB 50 and TB 100) and two non-structural fault states (OS 1500 and OS 2000). “TB 50” and “TB 100” refer to 50% and 100% tooth breakage in driving gear, respectively. Based on the baseline oil of 2600 mL, “OS 1500” and “OS 2000” refer to the reduction of 600 mL and 1100 mL of oil from GB1, respectively. Vibration signals and infrared images were collected under four different loads of 0%, 30%, 70% and 100% (L0, L30, L70 and L 100). For each load, vibration signals in each state were divided into 800 samples with 2048 data points. Four-hundred and eighty samples were randomly selected as tests, and the remaining 320 samples were used to train. Similarly, 480 infrared images were used to train, and the other 320 were used to test. The size of each infrared thermal image is 64 × 32. The details of the dataset are listed in Table 2.

### 4.2. Implementation Details

**Figure 3:**
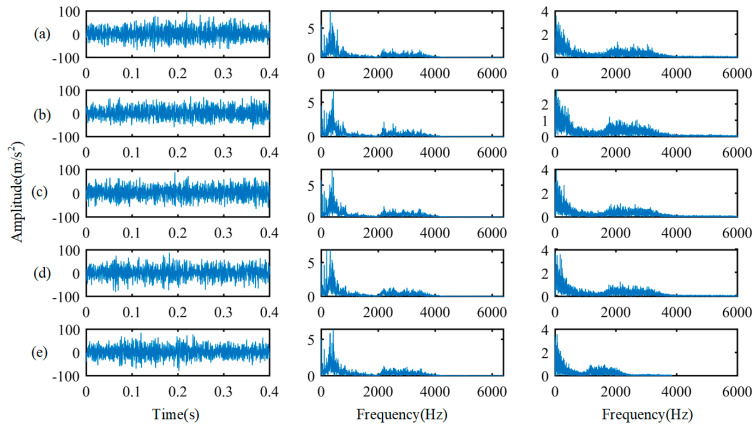
Raw signals, spectral distribution and squared envelope spectrum of different health states. (**a**) Normal; (**b**) TB 50; (**c**) TB 100; (**d**) OS 1500; (**e**) OS 2000.

At first, data-level fusion strategy is used to fuse infrared thermal images and vibration signals. The measured raw samples in five different health states are transformed to acquire frequency domain signals by FFT and CS2 by squared envelope spectrum in this study. Under L0 load, the three kinds of signal waveforms of different health states are shown in Figure 3. As shown in Figure 3, the left column is the measured raw signals, and the middle column and the right column are the corresponding spectral distribution and squared envelope spectrum, respectively. It can be observed that the time-domain characteristics and frequency domain characteristics of each health state are relatively similar and difficult to distinguish. Then, frequency domain signals and CS2 sequence are arranged into 2 × 64 × 32 formats.

**Figure 4:**
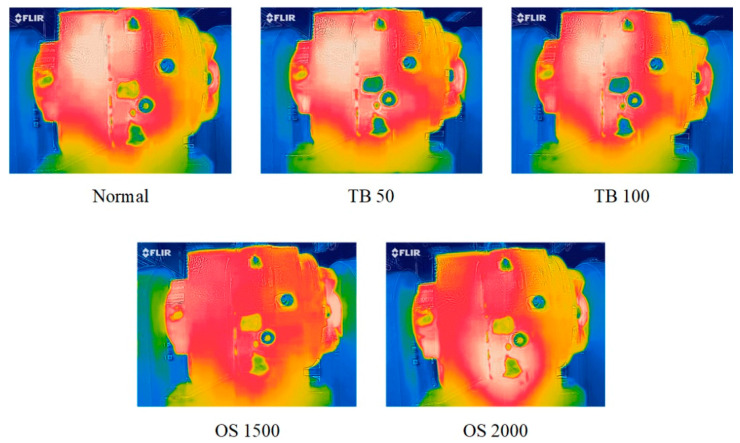
The infrared thermal image of different health states.

Each infrared thermal image has RGB channels, i.e., 3 × 64 × 32 formats. The collected infrared thermal images of each health state under L0 are shown in Figure 4. From Figure 4, we can observe that the images of normal, OS 1500 and OS 2000 are relatively similar, and the images of TB 50 and TB 100 are relatively similar. However, it is still very difficult to visually distinguish concrete health states. RGB channels of the infrared thermal image (3 × 64 × 32) will be combined with the frequency domain signals and CS2 (2 × 64 × 32), and the 5-channel fusion samples (5 × 64 × 32) are obtained to train DACNN.

In DACNN, a feature extractor, a domain discriminator and a state classifier were constructed, and the structures of those are listed in Table 3. The DACNN is trained by adversarial training using fusion data. In order to illustrate the robustness of the proposed method, multiple test tasks are designed. The concrete setting of different tasks and the results are listed in Table 4. It can be observed that the proposed method has good performance among the five test tasks, especially the accuracy of reaching 100.00% in T1, T3 and T4. In task T5, the transfer span is larger from load L0 to L100. The accuracy of the proposed method can still reach 96.67%. This suggests that the fusion of infrared thermal images and vibration signals to implement cross-domain fault diagnosis has good performance, and the result is relatively robust. 

### 4.3. Methods Comparison and Results Discussion

In this section, four methods are employed for comparison on the test tasks T1~T5 to illustrate the superiority of the proposed FDACNN. The details of compared methods are described as below.

The standard DANN method is utilized for comparisons [35]. Training data are sequences that convert fusion data into one dimension.

Another popular domain-adaptation method based on maximum mean discrepancy (DA-MMD) is applied for comparisons [36]. The train data and network structure used in DA-MMD are consistent with the proposed method.

The DACNN model was trained using single vibration signals (DACNN-SV). This model is trained using frequency domain signals and CS2 sequence (2 × 64 × 32 formats). 

The DACNN model was trained using single infrared thermal image (DACNN-SI). This model is trained using RGB 3-channels of infrared thermal images (3 × 64 × 32 formats).

**Figure 5:**
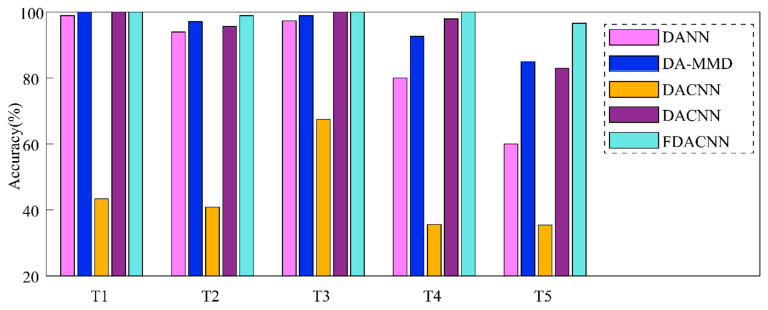
Bar diagram of results in different test tasks.

The results of different comparison methods are listed in Table 5. Meanwhile, in order to compare the results more intuitively, the bar diagram of results is shown in Figure 5. It can be observed that DACNN_SV has the lowest accuracy in five tasks: 47.38%, 40.88%, 67.44%, 35.56% and 35.44%, respectively. This is due to the fact that the vibration signal is not sensitive to two non-structural faults, which makes it impossible to classify, and the resulting accuracy is low. DACNN_SI has a good performance on T1 and T3, but it is not satisfactory in other test tasks; in particular, the accuracy of T5 is only 83%. It illustrates that infrared thermal images can be used to identify structural and non-structural failure states effectively, but they are susceptible to environmental interference. From the results of DANN and DA-MMD, it can be observed that the accuracy in all test tasks is lower than the proposed FDACNN. This is because two-dimensional data fusion can effectively maintain the fault information contained in the infrared image and vibration signal. Meanwhile, the adversarial domain adaptation network can enable the extracted extractor to extract target domain features that are easy to distinguish. 

**Figure 6:**
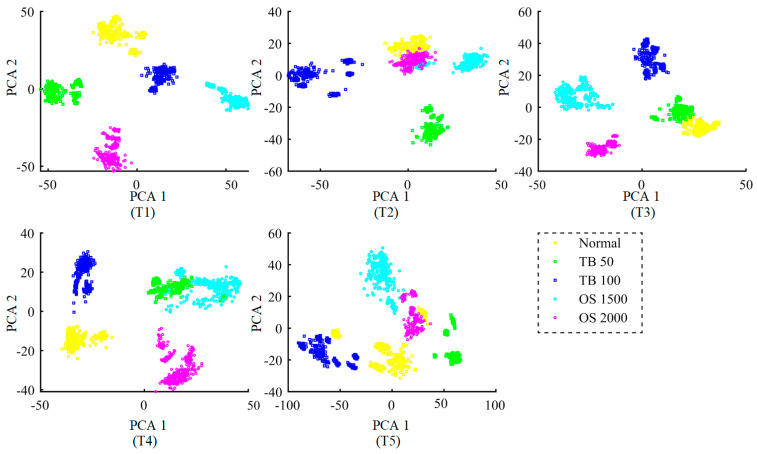
Feature visualization of different test tasks.

Additionally, in order to demonstrate the ability of the feature extractor to extract domain invariant features, principal analysis (PCA) is used to map extracted features into 2-dimensional space. Figure 6 shows 2-dimensional visualizations in different test tasks, in which PCA 1 and PCA 2 denote first and second principal components, respectively. It can be observed that the points with the same color are clustered in T1 and T3, and the point clusters of different colors are obviously isolated. In T2, T4 and T5, only a few points with the same color are confused, and most of them with the same color are relatively concentrated. Therefore, extracted features are relatively separable in all test tasks. It suggests that the trained feature extractor has the ability to extract distinguishable features from the unlabeled target domain fusion samples.

## 5. Conclusions

This study focuses on cross-domain fault diagnosis of gearboxes via multi-source heterogeneous data. Infrared thermal images and vibration signals are fused to characterize the health states of the gearbox, which can effectively recognize structural and non-structural faults. Moreover, the domain-adaptation neural network is trained via adversarial training using fusion data samples to extract the common transfer knowledge, called the FDACNN method. By performing this, the proposed FDACNN method can be used to recognize the unlabeled target domain samples of gearbox. For validation, the proposed FDACNN method is used to analyze gearbox multi-source heterogeneous data measured under various operating conditions. Moreover, we compare the FDACNN method with four other relevant methods to confirm its superiority in cross-domain fault diagnosis of gearboxes under various operating conditions. The results demonstrate that the proposed method obtains highest classification accuracy among four methods.

## Figures and Tables

**Table 1:** The detailed parameters of the infrared camera.

Parameters	Values
Alg type	PHE
Frame rate	25 fps
Temperate measurement range	−25 °C~260 °C
Environment temperature	18.9 °C
Thermal sensitivity	0.050 °C
Image resolution	384 × 288
Contrast	50
Brightness	50
Gain	2
Palette	rainbow

**Table 2:** 5 health states of gearbox.

Label	Health States	The Number of Training/Testing Samples
1	Normal	480/320
2	TB 50	480/320
3	TB 100	480/320
4	OS 1500	480/320
5	OS 2000	480/320

**Table 3:** The structures of features extractor, domain discriminator and states classifier.

Model	Layer	Filter Number	Size of Kernel	Output Size	Stride	Padding	Active Function
Features extractor	Conv2d 1	8	3 × 3	8 × 62 × 30	[1,1]	0	ReLU
	BN 1	8	-	8 × 62 × 30	-	-	-
	MaxPool2d 1	8	2 × 2	8 × 31 × 15	[2,2]	-	-
	Conv2d 2	16	3 × 3	16 × 29 × 13	[1,1]	0	ReLU
	BN 2	16	-	16 × 29 × 13	-	-	-
	MaxPool2d 2	16	2 × 2	16 × 14 × 6	[2,2]	-	-
	FC 1	-	-	680	-	-	-
	FC 2	-	-	300	-	-	ReLU
	FC 3	-	-	56	-	-	ReLU
	FC 4	-	-	28	-	-	ReLU
States classifier	FC	-	-	5	-	-	Softmax
Domain discriminator	FC	-	-	1	-	-	Softmax

**Table 4:** Result of different test tasks.

Tasks	Source Domain	Target Domain	Accuracy (%)
T1	L0	L30	100.00%
T2	L0	L70	98.98%
T3	L30	L70	100.00%
T4	L70	L0	100.00%
T5	L0	L100	96.67%

**Table 5:** The results of different comparisons methods.

Tasks	DANN	DA-MMD	DACNN_SV	DACNN_SI	Proposed FDACNN
T1	98.98%	100.00%	47.38%	100%	100.00%
T2	92.96%	97.12%	40.88%	95.69%	98.98%
T3	97.38%	98.97%	67.44%	100%	100.00%
T4	80.00%	92.69%	35.56%	97.94%	100.00%
T5	60.00%	85%	35.44%	83%	96.67%

## Data Availability

The data presented in this study are available on request from the corresponding author.
